# How deep is your recovery? Rethinking true motor recovery versus compensation in neurorehabilitation

**DOI:** 10.1097/MRR.0000000000000701

**Published:** 2026-04-27

**Authors:** Antonio Caronni, Maurizio Amadei, Viviana Rota, Michela Picardi

**Affiliations:** a Department of Neurorehabilitation Sciences, IRCCS Istituto Auxologico Italiano; b Department of Biomedical Sciences for Health, University of Milan; c Department of Neurorehabilitation Sciences, Casa di Cura Igea, Milan, Italy

**Keywords:** assessment, improvement, motor recovery, motor system, neurorehabilitation, restoration

## Abstract

Attempts to make a sharp distinction between ‘true’ recovery and compensation create ambiguity about the nature of motor improvement in neurological rehabilitation. This paper proposes a new framework to resolve this dichotomy: a stratified model where recovery is not a binary concept but is instead defined by its depth across the motor system’s multiple layers, from neurons to task performance. Our model frames the motor system as a hierarchy of causally linked strata, ranging from inner physiological layers (e.g. synaptic plasticity) to outer behavioural ones (e.g. task accomplishment). This framework allows motor improvement to be defined as a phenomenon that can occur at different layers, manifesting as a compensation–recovery gradient that propagates from inner-to-outer strata. The model’s central thesis is that the key question is not whether recovery is ‘true’, but how deep it is. We posit that a deeper recovery – one in which improvements in behavioural performance are underpinned by positive changes in inner physiological strata – constitutes a more complete and desirable clinical outcome. When comparing treatments with equivalent behavioural outcomes, those that also result in recovery in deeper physiological strata are superior and should be preferred. Therefore, this model directs neurorehabilitation research and practice toward interventions that also maximise recovery depth rather than focussing solely on behavioural outcomes. Assessing and demonstrating recovery depth requires that clinical psychometrics are complemented by quantitative metrics from instrumented movement analysis and neurophysiology, which can directly probe the inner, physiological strata of the motor system.

**Clinical Trials Registry:**

NA.

## Background

Rehabilitation, encompassing key interventions like physiotherapy, occupational therapy and speech therapy, is a cornerstone for motor-impaired individuals whose condition is caused by neurological disease [1,2].

In rehabilitation, diagnosis and treatment prescription are embodied in the processes of assessment and goal setting. The WHO’s International Classification of Functioning, Disability and Health (ICF) provides the standard framework for defining these goals [3,4]. According to the ICF, goals can be set at three distinct levels: addressing impairments of body functions and structures, overcoming limitations in activities, or enhancing participation in life situations.

**Box 1. Stratified model of recovery: problem, proposal, and takeaway**

**Problem:** The traditional dichotomy between ‘true’ recovery and compensation is conceptually ambiguous, as motor improvement typically involves both processes across different levels of the motor system.

**Proposal:** We propose a stratified framework conceptualising the motor system as a hierarchy of causally linked strata, from neuronal physiology to behavioural task performance. Recovery is defined as a measurable improvement in a motor variable within any specific stratum during task execution. For instance, improved hand spatial accuracy during an upper limb reaching task following stroke represents recovery at the end-effector stratum. If this improvement is underpinned by increased elbow extension, recovery also occurs at the deeper joint-kinematic stratum. Conversely, if end-effector improvement is achieved through increased trunk flexion without changes in elbow kinematics, recovery at the superficial stratum is associated with compensation at the kinematic level. This hierarchical logic extends to the deeper levels of neural circuitry and neuronal activity.

**Takeaway:** The clinical focus should shift from distinguishing between ‘true’ recovery and compensation to assessing ‘recovery depth’ – the degree to which behavioural improvements are underpinned by physiological restoration, ideally extending to the neuronal stratum. This assessment requires the integration of instrumented movement analysis and motor neurophysiology to probe the inner strata of the motor system.

To illustrate this framework, consider a sensory ataxic individual whose condition is due to multiple sclerosis, undergoing a balance training program. Following the ICF domains, a goal at the body functions level would be to improve balance reactions (e.g. standing on an unstable surface). At the activities level, this might translate into walking independently with a walker. Finally, at the participation level, the ultimate goal could be using this ability for a meaningful personal activity, such as shopping with her daughter. If the patient achieves these objectives, she is commonly said to have ‘recovered’. This common clinical description, however, raises the fundamental question that this paper seeks to address: What is motor recovery?

This paper addresses this question by proposing a new model of motor recovery for physical and rehabilitation medicine, built upon currently available evidence [5,6]. We argue that recovery should not be viewed as a monolithic event, but as a phenomenon that can occur across a hierarchy of strata within the motor system – from the neuronal to the behavioural. Within each stratum, recovery progresses along a continuous gradient and, upon reaching a critical threshold [7], these improvements propagate to the upper layers.

The model’s central thesis is that the most critical dimension for assessment is the depth of recovery. This perspective provides a framework for classifying recovery, guiding clinical choices, and justifying the integration of instrumented movement analysis and neurophysiology with traditional clinical scales in recovery assessment.

## Problem

### Two ways of getting better: recovering and compensating

The term ‘motor recovery’ is frequently used in neurological rehabilitation, though often with different and sometimes conflicting meanings [1]. At its core, the concept implies a return to a normal condition. However, the rehabilitation literature has historically split this idea into two contrasting categories: so-called ‘true’ recovery and compensation [8–10]. The first category, often considered the more desirable outcome, is referred to by terms such as intrinsic recovery, restoration, or simply recovery in its strictest sense. The second is known as compensation, adaptive recovery, or adaptation. This long-standing dichotomy has created significant ambiguity in both clinical and research settings.

### Task definition and the target of motor improvement

To illustrate this dichotomy, consider an acute stroke patient exhibiting severe upper limb impairment, which prevents them from performing a simple reach-to-grasp task (e.g. moving a glass on a table) with their affected arm. To succeed, the patient naturally uses their unaffected arm. In classic clinical terminology, his action is a classic example of compensation. As weeks pass, however, the patient regains sufficient function in the affected limb to perform the same task [11,12]. This development represents the other side of the dichotomy: recovery has occurred.

The same distinction between compensation and recovery applies to the recovery of walking. A patient unable to walk after a stroke might first achieve mobility by using a wheelchair; this is a clear form of adaptive recovery, as the broader goal of locomotion is met via a compensatory strategy used to overcome the loss of walking. If, over the following months, that same patient regains the ability to walk independently, the outcome is then classified as recovery.

These examples highlight a fundamental ambiguity: the distinction between recovery and compensation depends entirely on the precise definition of the task. In the reaching vignette, is the task simply ‘to move the glass’, or is it more specifically ‘to move the glass with the affected arm?’ The interpretation of the outcome changes completely depending on the answer. If the task is defined broadly (to move the glass), then using the unaffected arm is a successful instance of recovery of that general function. If the task is defined narrowly (to use the affected arm), the same action must be classified as a compensatory strategy – a new motor solution adopted to bypass the impairment [11,12]. Similarly, for a nonambulatory patient, using a wheelchair represents recovery of the general task of ‘locomotion’, but a compensation for the specific task of ‘walking’. This definitional subtlety is the foundation of the model proposed here.

## Position

### From kinematics to neurons: the relativism of recovery

**Fig. 1:**
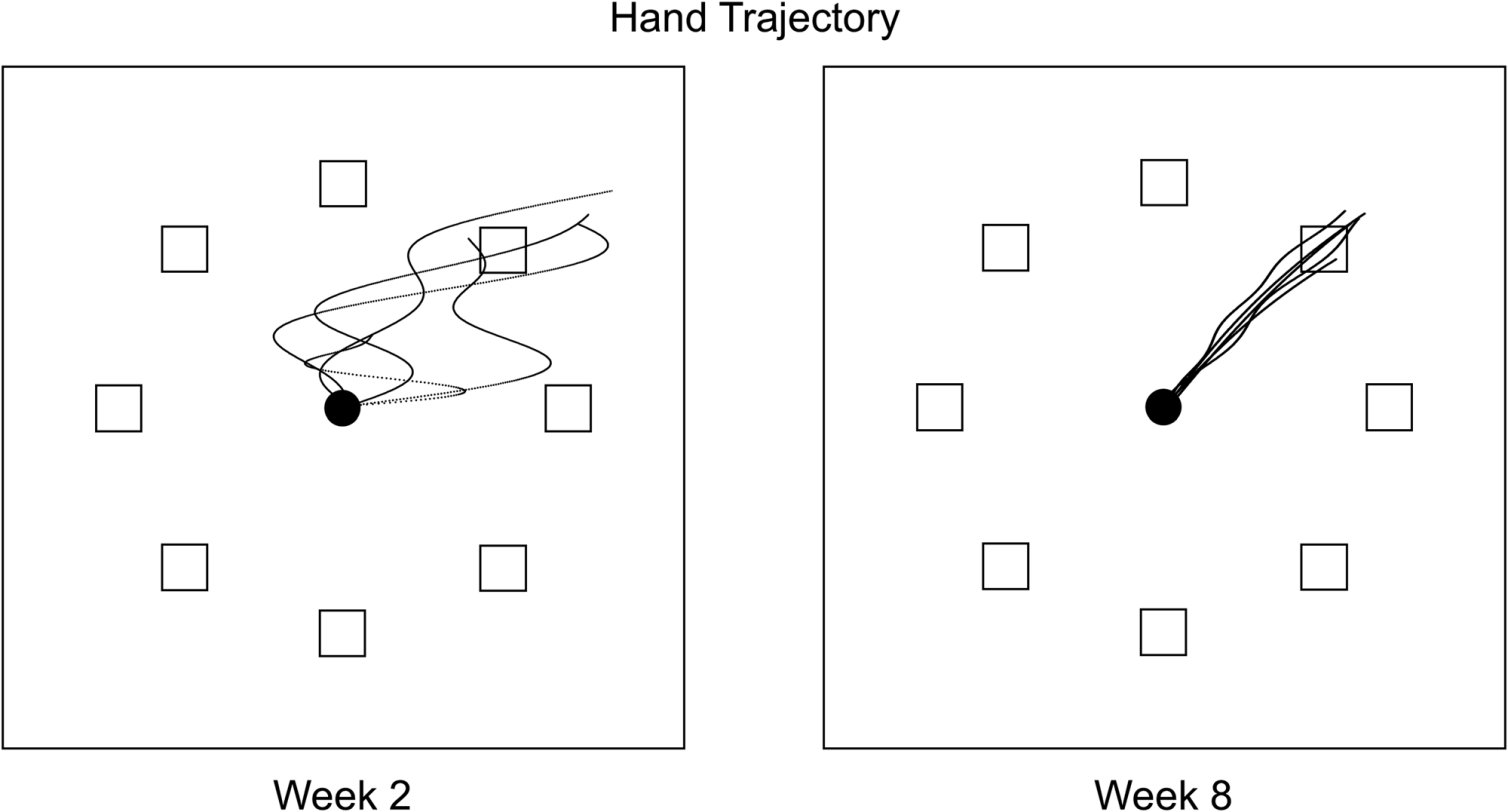
Simulated hand trajectories illustrating motor recovery after stroke. The figure shows simulated data from a hypothetical patient performing a tabletop reaching task. The patient moves their hand from a central starting position (black circle) to one of eight targets (squares). The panels contrast performance at two time points: two weeks poststroke (left) and eight weeks poststroke (right). Each panel displays five superimposed trajectories for movements toward the top-right target.

To visualise how the movement itself changes during recovery, Fig. 1 shows simulated hand trajectories from a hypothetical stroke patient performing a tabletop reaching task. The figure contrasts the patient’s performance at two time points: early after the stroke (two weeks) and later in the recovery process (eight weeks). For each condition, the superimposed trajectories of five repeated movements are displayed.

Early poststroke (Fig. 1, left panel), the patient’s movements are characterised by low precision and high variability. This variability is evident both in the final hand position across trials and within each individual movement, which shows multiple corrective sub-movements, that is, ‘turns’, instead of a direct path. Weeks later (right panel), significant improvements are apparent. The trajectories become smoother [13], precision increases, and trial-to-trial variability is markedly reduced. Movement speed typically increases as well. Taken together, these changes constitute a clear instance of recovery at the level of hand kinematics in the ‘reaching a target with the impaired arm’ task.

Let us now analyse this task from a different perspective, by shifting our focus from hand kinematics to a deeper stratum of the motor system: the joint mechanics (Table 1). The task remains the same: a seated individual ‘reaching for a glass with the affected arm’. In healthy subjects, this action is typically accomplished through a combination of arm elongation (shoulder flexion and elbow extension) and some degree of forward trunk flexion, although some individuals can complete the task using only arm movements if the object is within arm’s length [14].

**Table 1:**
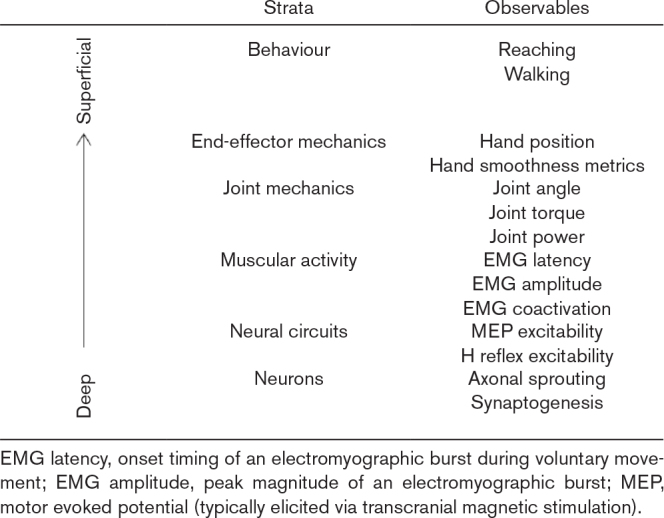
The proposed stratified model of the motor system, with example observables for each stratum

In contrast, stroke patients can accomplish the task by relying almost exclusively on forward trunk flexion, with negligible arm elongation [14]. This trunk movement pattern is a classic and clinically apparent compensation for the inability to extend the arm [15].

This clinical scenario leads to a crucial insight. For these patients, the recovery has occurred at the outer strata: the behavioural goal is met (the glass is moved) and the end-effector kinematics are effective (the hand reaches its target). At the deeper stratum of joint mechanics, however, the opposite is true. Here, no recovery of the typical arm movement has occurred. Instead, the excessive trunk flexion has compensated for the impaired arm kinematics, enabling the successful outcome at the higher levels. This reveals the central tenet of our model: the same behaviour can simultaneously represent recovery at one level and compensation at another.

This analysis illustrates a key principle: our interpretation of recovery versus compensation changes as we shift our analytical focus down through the motor system’s strata – from the behavioural goal, to end-effector kinematics, and finally to joint mechanics. This flexibility is possible because of a fundamental property of the nervous system: motor redundancy [16]. This principle holds that any given movement outcome (e.g. a hand reaching a target) can be achieved via a vast number of different combinations of joint rotations and muscle activations. It is precisely this redundancy that allows the system to discover novel compensatory strategies to bypass an impairment at one inner stratum while still achieving the goal at an outer one.

Moving one stratum deeper brings us to the level of muscular activity, which is typically measured with surface electromyography. Research on arm movement recovery in stroke, for example, has shown that disorganised patterns of muscle activation (often described as muscle synergies) can gradually normalise with treatment [17,18]. Within our model, this normalisation represents a clear instance of recovery at the muscular activity stratum, a level deeper than joint mechanics.

Descending further into our model, we reach the stratum of neural circuits, exemplified by the corticospinal system. After lesions such as stroke or spinal cord injury, corticospinal excitability is typically reduced [19]. This property can be measured noninvasively in humans using transcranial magnetic stimulation. Crucially, research demonstrates a direct link between motor skill improvement and increased excitability in the lesioned corticospinal system [20]. This neurophysiological restoration is a clear example of intrinsic recovery occurring at the neural circuit stratum.

The final and deepest stratum of the model is the cellular level, concerning the individual neurons themselves. This brings us to a fundamental question: do the cascading improvements observed across all the outer strata – from task performance down to neural circuit excitability – truly reflect an intrinsic recovery of the neurons that were lost?

### The deepest stratum: synaptic plasticity and the compensatory nature of recovery

To answer the previous question, let us frame it within a specific context: a chronic stroke patient (e.g. 6–12 months postinjury) with a stable, well defined lesion, such as an ischaemic lesion of the posterior limb of the internal capsule. In this phase, the early mechanisms of spontaneous biological recovery – such as the reduction of oedema or the resolution of diaschisis – are considered complete [21]. Furthermore, given the current state of medical science, we must assume that significant regeneration of lost neurons does not occur. This presents a crucial paradox, because it is also well established that meaningful motor recovery is still possible during this chronic period [2].

So, how is this recovery possible? The solution to the paradox lies in neuronal plasticity. The spared neurons that are still viable for the task can strengthen their action [22] by modulating synaptic activity and sprouting new connections.

This reveals a profound conclusion at the deepest level of our model. While this plasticity drives the recovery observed at higher strata (e.g. improved circuit excitability), the process itself is not a recovery of the original, lost tissue. At the cellular stratum, it is fundamentally a compensatory mechanism: the remaining healthy neurons adapt to make up for the neuronal loss. Therefore, at the neuronal level, what we call ‘recovery’ is always a form of compensation.

### A model of task- and stratum-specific improvement

The preceding analysis reveals the two foundational principles of our model. First, any discussion of recovery is meaningless without a precise definition of the task. Second, recovery is always the recovery of something: a specific, measurable variable.

We therefore apply these principles to our stratified framework (Table 1). We define recovery as the restoration of a variable of a given stratum to within a normative range during a specific task. Compensation, in turn, describes the modifications adopted within one stratum to overcome the lack of recovery in a specific variable. These compensatory modifications often serve to enable the recovery of a variable in a different, typically higher, stratum.

Applying our new definitions to the arm reaching example in stroke helps clarify this interplay. At the inner stratum of joint mechanics, there was no recovery of arm movement. Instead, the patient’s trunk kinematics compensated for this impairment. This compensation at a deeper level, in turn, enabled the intrinsic recovery of the hand’s movement at the outer, end-effector stratum.

This illustrates a core tenet of our model: compensation within one stratum is the mechanism that can enable recovery in another. Therefore, recovery and compensation are not mutually exclusive but rather coexist as interconnected processes along a continuum.

To formalise these principles, our model conceptualises the motor system as a hierarchy of six strata, ranging from the outer behavioural stratum to the inner neuronal one (Table 1). Each stratum contains specific variables, or ‘observables’, that can be quantified. For instance, upper limb dexterity is an observable at the behavioural level, whereas corticospinal excitability (measured with transcranial magnetic stimulation) is an observable at the deeper neural circuitry level. The strata are causally linked from the inner to the outer, and the behavioural stratum itself can be further subdivided according to the ICF domains of function, activity, and participation.

### Threshold dynamics and the non-linearity of multistratum recovery

Beyond guiding choices between existing therapies, a second fundamental reason exists for assessing the inner strata: to better understand recovery mechanisms for the purpose of developing novel treatments.

As our model posits, the strata are causally linked from the bottom up; for instance, improvements at the neuronal stratum (e.g. synaptic plasticity offsetting neuronal depletion) can lead to increased excitability at the neural circuit stratum. Crucially, however, this causal relationship is not always linear or immediately apparent. An improvement in a deep stratum, such as increased corticospinal excitability, does not automatically translate into better muscle activation or kinematics [23]. For a change in an inner stratum to propagate outwards and manifest as a visible improvement in an outer stratum, it must first cross a critical threshold [7].

This threshold mechanism, supported by both experimental evidence [24] and clinical intuition, can be explained by two nonexclusive factors.

First, an improvement in an inner stratum must be of sufficient magnitude to cause an effect in a more superficial stratum. This is analogous to lifting a weight: any increase in muscular force is inconsequential until it is large enough to overcome the object’s mass.

Second, a single behavioural outcome often relies on the convergence of multiple variables from deeper strata. For example, upper limb dexterity at the behavioural level only emerges if improvements occur in both movement precision and smoothness at the underlying kinematic stratum.

This causal, bottom-up structure of recovery has a profound implication for therapeutic development. By studying the inner strata, clinicians and researchers can identify and measure ‘sub-threshold’ improvements – changes that have occurred but are not yet large enough to be clinically apparent. A deep understanding of these underlying mechanisms can be the key to designing novel treatments (e.g. [25].) that can amplify these nascent changes, push them across the threshold, and ultimately produce robust and meaningful recovery at the behavioural levels.

In the proposed model, strata are conceptualised as superimposed and discrete, with improvements within a specific stratum manifesting in more superficial layers only after surpassing a critical threshold. This discretisation and the associated threshold mechanism facilitate expository clarity and reflect the intrinsic complexity of the recovery process, characterised by nonlinear dynamics and unpredictability [7]. However, within each stratum, the underlying recovery is continuous and progressive.

Returning to the previous examples, within the neuronal stratum, the number of synapses and the strength of their connections increase progressively. Once this continuous compensatory process reaches a sufficient magnitude – crossing the critical threshold [7] – it becomes apparent in the adjacent upper stratum as the recovery of a higher-order function (e.g. circuit excitability, dexterity).

## Synthesis

This section aims to situate the proposed framework within existing theories and competing viewpoints. In doing so, it also explores the broader implications of this stance for rehabilitation research and clinical practice.

### Reconciling inner strata with meaningful clinical goals

A focus on recovery within the inner strata of our model may seem to contradict the current consensus on goal setting in motor rehabilitation [5]. This consensus rightly prioritises meaningful, patient-centred outcomes, such as reducing activity limitations and enhancing participation. From this perspective, the primary clinical aim is not to restore ‘perfect’ kinematics or normalise motoneuron recruitment per se, but to reduce a person’s overall disability. In the context of stroke, for example, motor rehabilitation is typically initiated with the explicit goal of enhancing a patient’s independence and participation [5].

This focus on disability outcomes is embodied in the widespread recommendation of task-oriented training [26]. This approach emphasises the practice of meaningful actions drawn from real-life situations – such as components of the activities of daily living – over rote, repetitive exercises [27]. For instance, a patient will practice a reaching movement rather than performing ‘three sets of 12 reps’ of isolated elbow extensions [28].

We fully agree with this patient-centred perspective. A therapy that produces ‘recovery’ in an inner stratum (e.g. improving corticospinal excitability) but provides no benefit to a person’s daily activities is clinically irrelevant. Such an approach would be analogous to an antihypertensive drug that normalises blood pressure but fails to reduce the risk of stroke [29].

Instead, our model’s contribution is that when two treatments yield equally good behavioural outcomes, the therapy that also promotes recovery in the deeper, physiological strata is unequivocally superior. A recovery that is not just apparent at the functional level but is also underpinned by physiological restoration is more complete, and thus preferable [29,30].

To illustrate, consider a therapy that not only reduces disability at the behavioural stratum (e.g. improving Barthel Index scores) but also enhances upper limb dexterity at a deeper stratum (e.g. improving Fugl–Meyer Assessment scores). This treatment would be superior to one that achieves the same reduction in disability but without corresponding improvements in dexterity [24].

Therefore, assessing the inner strata is not a mere academic exercise. It is a clinical necessity to choose interventions that provide the fullest and most robust recovery.

### Generalising the framework: from focal lesions to neurodegenerative disorders

A key aspect to consider is the scope of the model’s applicability. Much of the literature on the distinction between intrinsic and adaptive recovery – and, accordingly, the examples in this paper – has focussed on motor recovery after stroke, particularly in the context of the pyramidal syndrome. However, the stratified framework we propose is intentionally general and is likely applicable to a wide range of neurological conditions beyond focal lesions.

Neurodegenerative diseases, for instance, provide a particularly compelling area for the model’s future application. While the regeneration of lost neural tissue is not considered a significant mechanism for recovery after a stable focal injury like stroke – though it remains an important avenue of research [31] – the situation is different for progressive neurodegenerative diseases. Certain treatments, such as endurance training in Parkinson’s disease, are thought to have neuroprotective effects that slow the rate of neuronal loss [32–34].

According to our model, this slowing of degeneration constitutes intrinsic neuronal recovery following treatment. The rationale is that the net result is the same as that of regeneration: relative to the disease’s expected trajectory, a greater number of neurons remain viable.

The model is also readily applicable to gait recovery in Parkinson’s disease. Following treatment, a patient might successfully increase their overall gait speed, which represents an intrinsic recovery of that variable [35]. An analysis at the deeper, end-effector stratum, however, could reveal that this was achieved solely by increasing step frequency, with no improvement in the characteristically short step length [36]. In this scenario, the core Parkinsonian gait impairment has not been restored. Instead, the increased cadence is a compensatory strategy at one stratum that enables the successful recovery of gait speed at a higher one.

### Comparison with previous models of recovery

The goal of this paper is not to provide an exhaustive systematic review of terminology. Rather, our aim is to propose a new conceptual model and situate it relative to what is arguably the most influential prior work in this domain.

The concept of a layered approach to recovery is not entirely new, and the key benchmark for comparison is the perspective paper by Levin, Kleim, and Wolf on stroke recovery [37]. Their model also distinguished three levels (neuronal, motor performance, and functional) within the ICF framework [4] and, like ours, emphasised the use of movement analysis to better understand the recovery process.

However, beyond these foundational similarities, our model diverges from this earlier work on three fundamental points.

The first point of divergence is granularity. Our model offers a more detailed, multistratum hierarchy compared to the three levels of Levin *et al*.

Second, and more critically, is our definition of recovery. We propose that intrinsic recovery is not an all-or-nothing event but a process that can occur in any stratum, defined simply as a measurable improvement in a given variable. For instance, a patient resuming the ability to eat autonomously represents a ‘true’ recovery at the activities stratum. This contrasts sharply with the stricter, holistic definition of Levin *et al*., for whom recovery would require that the same task be performed ‘in the same manner as healthy controls’ ([37] p. 317).

The third fundamental difference concerns the philosophy of measurement. Levin *et al*. suggest the use of measures that assess multiple levels simultaneously, such as tests combining task success (a behavioural variable) with movement quality (a kinematic variable). We argue, in contrast, that a valid measure must be unidimensional and belong to a single stratum. Measures that cross-strata risk multidimensionality, a flaw that violates fundamental measurement principles [38].

A key advantage of our model is its practical applicability. The assessment process is straightforward: an investigator selects a specific task, a stratum of interest, and a measurable variable. Recovery is demonstrated if that variable returns to within a normative range. The crucial question then becomes not if recovery occurred, but how deep it went.

In contrast, defining recovery in holistic terms – that is, as the simultaneous restoration of all movement variables to match a healthy control’s pattern – makes it a concept that is difficult, if not impossible, to verify experimentally.

Central to this framework is the tenet that assessing inner strata is a clinical necessity for selecting interventions that maximise recovery depth. The requirement to investigate recovery within the deeper strata of the motor system has been previously noted by other authors.

For instance, the effects of interventions primarily targeting the outermost strata (e.g. the activities stratum) have been discussed in relation to recovery within deeper strata. Specifically, it has been observed that reliance on the nonparetic limb following stroke, for example, to recover an activity, can impede recovery within the deeper strata governing the kinematics of the paretic upper limb, as well as the innermost strata concerning neural circuitry in the ipsilesional hemisphere [39,40]. This example illustrates why evaluating deeper strata is not merely theoretical but clinically necessary: interventions that appear successful at the activities level may in fact constrain recovery at motor and neural levels.

The call for the development of kinematic and kinetic methods in clinical settings to complement clinical scores [41] aligns with the proposed framework and the necessity to investigate recovery within the deeper strata of the motor system. The emphasis on motor coordination quality during rehabilitation [41] reinforces the requirement to analyse recovery within strata deeper than the purely behavioural level measured by clinical scales.

Within the discourse on stroke recovery and compensation, global task-success metrics (endpoint measures) have been contrasted with kinematic analysis, asserting that only the latter enables the differentiation between recovery and compensatory patterns [10]. This is consistent with the proposed framework, wherein improvement at a superficial stratum (e.g. the end-effector) may be underpinned by either compensation or recovery at deeper strata (e.g. joint kinematics) [10].

The need to develop rehabilitation interventions that facilitate recovery within the deep strata of the motor system (specifically at the neuronal level) has also been underscored within the recovery versus compensation discourse.

For instance, the necessity of interventions targeting improvements within neural circuitry (which underpin the restoration of impaired functions) has been emphasised [42]. This perspective implicitly recognises at least two hierarchically organised strata: the neural (deep) and the behavioural (superficial). The need for rehabilitation therapies grounded in biological rationales has been emphasised, based on the continuum between neuronal-level mechanisms and behavioural outcomes [42].

Consistent with the requirement that recovery engage the deeper strata of the motor system, poststroke clinical and research perspectives (representing a paradigm shift) have proposed prioritising impairments as therapeutic targets rather than focusing on activities or quality-of-life outcomes [10]. This shift is predicated on the fact that impairment provides a more accurate representation of intrinsic biological repair mechanisms [10]. This approach inherently targets deep recovery, extending to the underlying biological strata.

Finally, within the established literature on recovery mechanisms, recent discourse suggests a fundamental reassessment of poststroke neuroplasticity. Conventional theory posits that following a vascular lesion, surviving neurons undergo dendritic sprouting and synaptogenesis. In contrast, an alternative perspective suggests that endogenous recovery mechanisms are not sufficiently robust to generate de novo synaptic connections. Instead, recovery (and associated phenomena such as cortical map reorganisation) may occur via the unmasking and strengthening of latent connections [22]. Critically, the specific physiological mechanism does not invalidate the proposed framework. The unmasking of silent connections represents a compensatory mechanism at the neuronal stratum that, if sufficiently intense, exceeds the threshold for recovery observed in more superficial strata.

### Assessing the depth of recovery

The proposed framework provides a systematic methodology for assessing the depth of recovery, with direct implications for rehabilitation research and clinical practice.

Assessing recovery requires a benchmark, but defining the ‘normal’ state to which a patient should return is complex. Ideally, true recovery would mean achieving the motor state the individual would have been in had the disease never occurred. This personal, counter-factual state is, however, empirically inaccessible.

Therefore, clinical practice and research rely on a pragmatic solution: comparing a patient’s performance against normative data from a healthy reference population. From this perspective, recovery is quantified as the process of approaching and entering this normative range. Establishing these normative boundaries is thus the foundational step for any objective assessment of recovery.

The assessment of motor recovery, therefore, becomes a process of quantitatively comparing a patient’s variable against a normative value. This comparison can be made at any stratum of the motor system, from behavioural disability down to the excitability of spinal reflexes. Quantifying the variables within these diverse strata requires specific technologies, ranging from questionnaires to instrumented movement analysis and neurophysiological techniques [43].

At the outermost behavioural stratum, assessment relies on psychometrics. Questionnaires are the primary tools for quantifying latent variables such as disability, arm dexterity, or locomotor ability. To probe the deeper strata of joint and end-effector mechanics, instrumented movement analysis is necessary. These technologies provide objective measures of kinematic and dynamic variables [44]. Finally, assessing the deepest neural circuit and neuronal strata requires neurophysiological techniques. Methods such as spinal reflex conditioning and transcranial magnetic stimulation allow for the direct measurement of the excitability of motor pathways [23,45].

These three approaches are not in competition; they are complementary. A comprehensive assessment of recovery depth (from the neuron to the patient’s lived experience) is only possible through their thoughtful integration.

## Limitations

A primary limitation of the model as presented is its emphasis on inner-to-outer causality. While changes at the neuronal level are requisite for new movements, the reverse causal pathway is a fundamental principle of neuronal plasticity. It is well established, for example, that the repeated practice of a motor task (an event at the outer behavioural stratum) is precisely what induces plastic synaptic changes at the inner neuronal stratum [46]. These top-down feedback connections remain to be fully explored within our framework and represent a key direction for future development.

Specifically, the model is predicated on a bottom-up progression across strata (e.g. compensation, recovery, recovery). For instance, the unmasking of latent connections (a compensatory mechanism for neuronal loss) supports an increase in corticospinal excitability (recovery), which in turn enables greater elbow extension during upper limb reaching (recovery of joint kinematics). However, nonsequential interactions between strata, such as a recovery–compensation–recovery sequence, are also plausible. In particular, improved corticospinal excitability might not be sufficient to restore full elbow kinematics, leading to the coexistence of neuronal recovery and kinematic compensation. Nevertheless, such neuronal improvement could still manifest as recovery in higher-order strata, such as increased end-effector precision (e.g. the hand in reaching movements). This suggests that compensation and recovery can be intertwined across hierarchical levels. The intriguing possibility that compensation may, in specific circumstances, facilitate intrinsic recovery has been previously put forward [39].

A second limitation is the paper’s exclusive focus on neurological diseases. The model’s principles, however, can be readily extended to motor conditions with a nonneurological origin, such as musculoskeletal disorders. For instance, consider a knee arthrosis patient who, after a steroid injection, regains the ability to climb stairs. According to our model, this represents an intrinsic recovery at the behavioural stratum. Yet, at the deeper tissue stratum, no recovery of the damaged cartilage has occurred. Instead, the drug’s pain-relieving effect allowed for compensating for the underlying joint pathology.

On a more granular level, the six strata presented in our model are not meant to be exhaustive, as their core logic can be extended to deeper levels of biological organisation. Our framework’s deepest level is the neuron, but neurons themselves are complex systems composed of sub-cellular components such as synapses, which in turn rely on organelles like mitochondria.

## Future directions

By highlighting the model’s recursive structure, the previous section points directly to future directions. This feature enables the extension of the framework to deeper levels of biological organisation and provides a foundation for further empirical investigation.

Consider a hypothetical disease where mitochondrial dysfunction impairs synaptic transmission [47]. A therapy that restores mitochondrial health could produce an intrinsic recovery at both the organelle and the synaptic strata. In contrast, a different therapy that improves synaptic transmission while bypassing the mitochondrial defect would represent an intrinsic recovery at the synaptic stratum, enabled by a compensatory mechanism at the deeper, organelle level.

Another significant future direction is to employ this stratified model as a clinical framework to guide patient assessment, treatment planning, and progress monitoring. To illustrate, consider the case of an incomplete cervical myelopathy patient (ASIA C). A clinician could use the strata to guide the assessment in a top-down manner. A problem at the outer behavioural stratum (for instance, a reduced Barthel Index score due to the need for assistance in transfers) could be traced to an impairment at the deeper activity stratum, such as an inability to perform a supine-to-sit transition. Deeper investigation might reveal that this, in turn, is caused by impairments at the muscular activity stratum: a combination of abdominal muscle weakness and spasticity in the hip adductors that is exacerbated by the manoeuvre. In this way, the model serves to formalise and make explicit the top-down diagnostic reasoning that is often a hallmark of expert clinical practice.

Conversely, treatment planning could proceed from the bottom up. Interventions could target the muscular stratum directly, for example, by using botulinum toxin to manage spasticity, complemented by passive mobilisation and stretching. At the adjacent, higher stratum of joint mechanics, a progressive resistance training program could be prescribed to strengthen the trunk flexor muscles.

Ongoing reassessment would then verify two things: first, that the treatments produced an intrinsic recovery in the targeted variables (e.g. reduced spasticity, increased strength), and second, whether these improvements at the inner strata have successfully propagated upwards to the higher levels.

This framework also provides a logical basis for integrating other modalities, such as non-invasive brain stimulation or pharmacological agents, which are designed to modulate plasticity at the deepest neuronal and circuit strata [45,48].

## Conclusions

This paper has proposed a conceptual model that reframes the debate on motor recovery. We argue that there is no ‘intrinsic’ or ‘adaptive’ recovery per se. Instead, the concept of recovery is always relative and must be defined by three essential components: (a) a specific variable; (b) a given stratum of the motor system; and (c) the motor task during which the recovery is assessed. Consequently, any scientifically or clinically meaningful statement about recovery must explicitly declare these three conditions.

Our model conceptualises the motor system as a hierarchy of stacked strata, from the inner neuronal level to the outer behavioural level, which are linked by a bottom-up causality: changes in deeper layers produce effects in the more superficial ones.

At the deepest, neuronal stratum, recovery is almost always compensatory, as surviving neurons adapt to make up for cell loss. Crucially, it is this foundational act of compensation that enables a cascade of genuine intrinsic recoveries across the more superficial strata – from restored circuit excitability, through improved muscle activation, all the way to effective task performance.

The clinical and scientific utility of this model is therefore two-fold. First, assessing the inner strata allows clinicians to select, from among treatments with similar behavioural outcomes, the one that provides a deeper and thus fuller recovery. Second, understanding the sub-threshold mechanisms at these deep levels provides the necessary insights to design the next generation of novel therapies.

Perhaps the most striking conclusion from our model is that an improvement in a measure like the Barthel Index [49] is, by definition, an intrinsic recovery of the variable we call ‘disability’. This may sound paradoxical, as it conflicts with the conventional clinical dichotomy between ‘intrinsic’ and ‘adaptive’ recovery.

This paradox, however, is revealing. It underscores that the traditional dichotomy is ultimately a matter of semantics. The critical question is not what label we use for recovery, but how deep it goes. The ultimate goal for both clinicians and researchers should be to understand this depth and to develop therapies that make a patient’s recovery as profound and complete as possible.

## Acknowledgements

This work was funded by the Italian Ministry of Health (Ricerca Corrente). The conceptual reflections presented in this manuscript provided the theoretical framework for the SASHA study (24C303; Trial registration: NCT06311526).

### Conflicts of interest

Antonio Caronni serves as an Associate Editor for the International Journal of Rehabilitation Research. For the remaining authors, there are no conflicts of interest.
